# Targeting lentiviral vector to specific cell types through surface displayed single chain antibody and fusogenic molecule

**DOI:** 10.1186/1743-422X-7-35

**Published:** 2010-02-11

**Authors:** Yuning Lei, Kye-Il Joo, Jonathan Zarzar, Clement Wong, Pin Wang

**Affiliations:** 1 Mork Family Department of Chemical Engineering and Materials Science, University of Southern California, Los Angeles, CA 90089, USA

## Abstract

**Background:**

Viral delivery remains one of the most commonly used techniques today in the field of gene therapy. However, one of the remaining hurdles is the off-targeting effect of viral delivery. To overcome this obstacle, we recently developed a method to incorporate an antibody and a fusogenic molecule (FM) as two distinct molecules into the lentiviral surface. In this report, we expand this strategy to utilize a single chain antibody (SCAb) for targeted transduction.

**Results:**

Two versions of the SCAb were generated to pair with our various engineered FMs by linking the heavy chain and the light chain variable domains of the anti-CD20 antibody (αCD20) via a GS linker and fusing them to the hinge-CH2-CH3 region of human IgG. The resulting protein was fused to either a HLA-A2 transmembrane domain or a VSVG transmembrane domain for anchoring purpose. Lentiviral vectors generated with either version of the SCAb and a selected FM were then characterized for binding and fusion activities in CD20-expressing cells.

**Conclusion:**

Certain combinations of the SCAb with various FMs could result in an increase in viral transduction. This two-molecule lentiviral vector system design allows for parallel optimization of the SCAb and FMs to improve targeted gene delivery.

## Introduction

Gene therapy is the introduction of a functional gene into a dysfunctional cell for a therapeutic benefit. To date, viral vectors remain the most commonly used gene delivery vehicles due to their high transduction efficiencies [1,2]. In particular, lentiviral vectors represent one of the most effective gene delivery vehicles as they allow for stable long-term transgene expression in both dividing and non-dividing cells. In order to expand the targeted specificity of viral vectors beyond their natural tropism, numerous studies have been focused on pseudotyping lentiviral vectors with envelope glycoproteins derived from other viruses, such as the glycoprotein from vesicular stomatitis virus (VSVG) [3,4]. However, since the VSVG is thought to recognize a ubiquitous membrane phospholipids instead of a unique cellular receptor, pseudotyping generates vectors with broad specificities [5,6]. To mitigate this off-target effect, previous attempts have been devoted to engineer the viral glycoprotein to recognize a specific cellular target by insertion of ligands, peptides, or antibodies [7-16]. Another approach involves bridging the viruses and the targeted cell with ligand proteins or antibodies [17-20]. However, these modifications to the surface glycoprotein appear to perturb the natural fusion function of the glycoprotein, resulting in a reduction of transduction efficiency.

Recently, our lab has developed a strategy to target lentiviral vectors to specific cell types by incorporating a surface antibody specific to CD20 antigen and a fusogenic molecule (FM) as two distinct molecules [21]. Kielian and co-workers reported several versions of the Sindbis virus glycoprotein that were less dependent on cholesterol for transduction [22]. We applied these mutations (E1 226) to the binding defective Sindbis glycoprotein and observed that they were able to enhance transduction efficiency when paired with an anti-CD20 antibody (αCD20) [23]. In this study, we report our attempt to utilize a single chain antibody (SCAb) to pair with a FM for targeting lentiviral vectors. Our SCAb is composed of variable domains of the heavy and light chains of αCD20, linked by a GS linker and fused to a hinge-CH2-CH3 region of human IgG. To anchor the SCAb onto the viral surface, we conjugated the SCAb with either the HLA-A2 transmembrane domain (SC2H7-A2) or the VSVG transmembrane domain (SC2H7-GS). We demonstrated that the lentiviral vector enveloped with either of these antibody configurations could achieve targeted transduction to CD20-expressing cells. We also compared the targeted transduction efficiency and the binding avidity of both versions of the SCAb and investigate the molecular roles of the displayed proteins in mediating lentiviral transduction.

## Results

### Construction of SCAb for targeting

**Figure 1:**
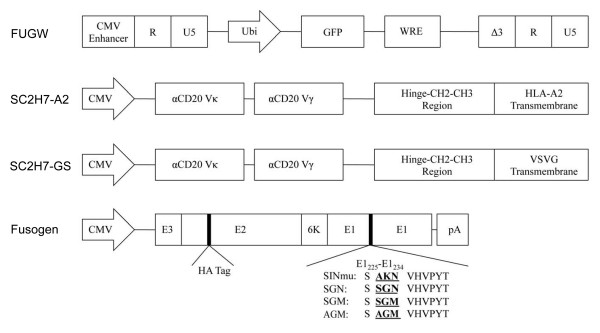
**Schematic representation of key constructs in this study**. These constructs include a lentiviral backbone vector FUGW, fusogenic molecule (FM) derived from Sindbis virus glycoprotein, membrane-bound single chain antibody against the CD20 antigen with either a HLA-A2 transmembrane domain (SC2H7-A2) or a VSVG transmembrane domain (SC2H7-GS). CMV enhancer: the enhancer element derived from human cytomegalovirus; GFP: enhanced green fluorescence protein; Ubi: the human ubiquitin-C promoter; WRE: woodchuck responsive element; CMV: human cytomegalovirus immediate-early gene promoter; αCD20 Vκ: the variable domain of the kappa chain of the mouse anti-CD20 antibody; αCD20 Vγ: the variable domain of the gamma chain of the mouse anti-CD20 antibody; CH2-CH3 region: the CH2-CH3 region of the human IgG1 antibody; HLA-A2 transmembrane domain: the transmembrane domain of the HLA-A2 protein; VSVG transmembrane domain: the transmembrane domain of the VSVG protein; E3: the leading peptide of Sindbis virus glycoprotein; E1: the E1 protein of Sindbis virus glycoprotein for mediating fusion; E2: the E2 protein of Sindbis virus glycoprotein for binding to cellular receptor; HA tag: 10-amino acid epitope sequence of hemagglutim; pA: polyadenylation signal.

We have previously demonstrated that targeting lentiviral vectors can be generated by co-transfecting producer cells with a lentiviral vector backbone plasmid, FUGW, a plasmid encoding an antibody's heavy and light chains, a plasmid encoding antibody accessory proteins, and a plasmid encoding a FM, along with lentiviral packaging plasmids [21,24]. In this report, we wanted to expand the targeting strategy by pairing FMs with SCAbs. To generate the SCAb for this study, we first PCR-amplified the light chain and heavy chain variable regions of the αCD20 and linked them with a GS linker. To allow for the formation of disulfide-linked dimmers to stabilize the SCAb, the hinge-CH2-CH3 region of the human IgG was fused to the heavy chain variable region [25-28]. To anchor the SCAb, the HLA-A2 transmembrane domain or the VSVG transmembrane domain was added to the C-terminal and the resulting constructs were designated as SC2H7-A2 and SC2H7-GS, respectively (Fig. 1).

### Production of lentiviral vectors

**Figure 2:**
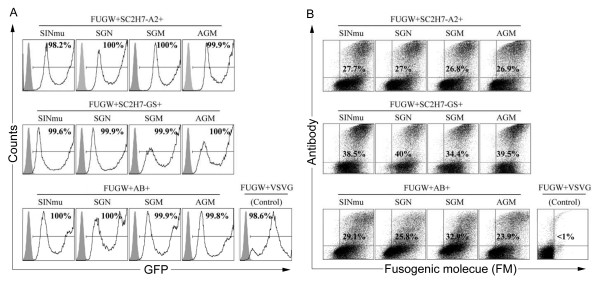
**Co-transfection of virus-producing cells to generate targeting lentiviral vectors**. 293T cells were transiently transfected with, FUGW, pSC2H7-A2, pFM and the other standard packaging plasmids (pMDLg/pRRE and pRSV-Rev) to make FUGW/SC2H7-A2/FM. Antibody construct pSC2H7-GS was used to generate FUGW/SC2H7-GS/FM. An isotype control antibody construct pAB was used in the transfection to produce non-targeting lentiviral vector FUGW/AB/FM. Transfection with plasmids encoding VSVG was used to generate a control vector, FUGW/VSVG. (A) FACS analysis of GFP expression on transfected cells. Solid line, analysis on transfected 293T/CD20 cells; shaded area, analysis on 293T cells. (B) Analysis of co-expression of the FM and antibody on gated GFP-positive cells. FM was stained using an anti-HA antibody and antibody was stained using an anti-human IgG antibody.

We generated SCAb-bearing lentiviral vectors (FUGW/SC2H7-A2/FM or FUGW/SC2H7-GS/FM) by co-transfecting 293T cells with the lentiviral backbone plasmid FUGW, a FM-encoding plasmid (SINmu, SGN, SGM, or AGM), and a plasmid encoding the described SCAb (pSC2H7-A2, or pSC2H7-GS) along with other necessary packaging plasmids (Fig. 1). Independently, a lentiviral vector bearing an isotype control antibody, pAB and a FM was produced as a non-target control. Furthermore, we included a VSVG-pseudotyped lentiviral vector, FUGW/VSVG as an additional positive control since VSVG-carrying viral vectors are known to transduce a variety of different cell types [4]. As shown in Fig. 2A, FACS analysis of transfected, virus-producing 293T cells showed that virtually all of the cells were able to be transfected with the viral backbone plasmid FUGW. Among the GFP-positive cells, roughly 25% to 40% of the producer cells were positive for both the antibody and the FM (Fig. 2B). As expected, transfection with VSVG as the envelope protein showed no expression of the FM and the SCAb. The similar levels of transfection and expression of the four FMs suggests that they could be incorporated into the lentiviral surface with similar efficiency.

### Incorporation of SCAb and FM onto lentiviral vectors

**Figure 3:**
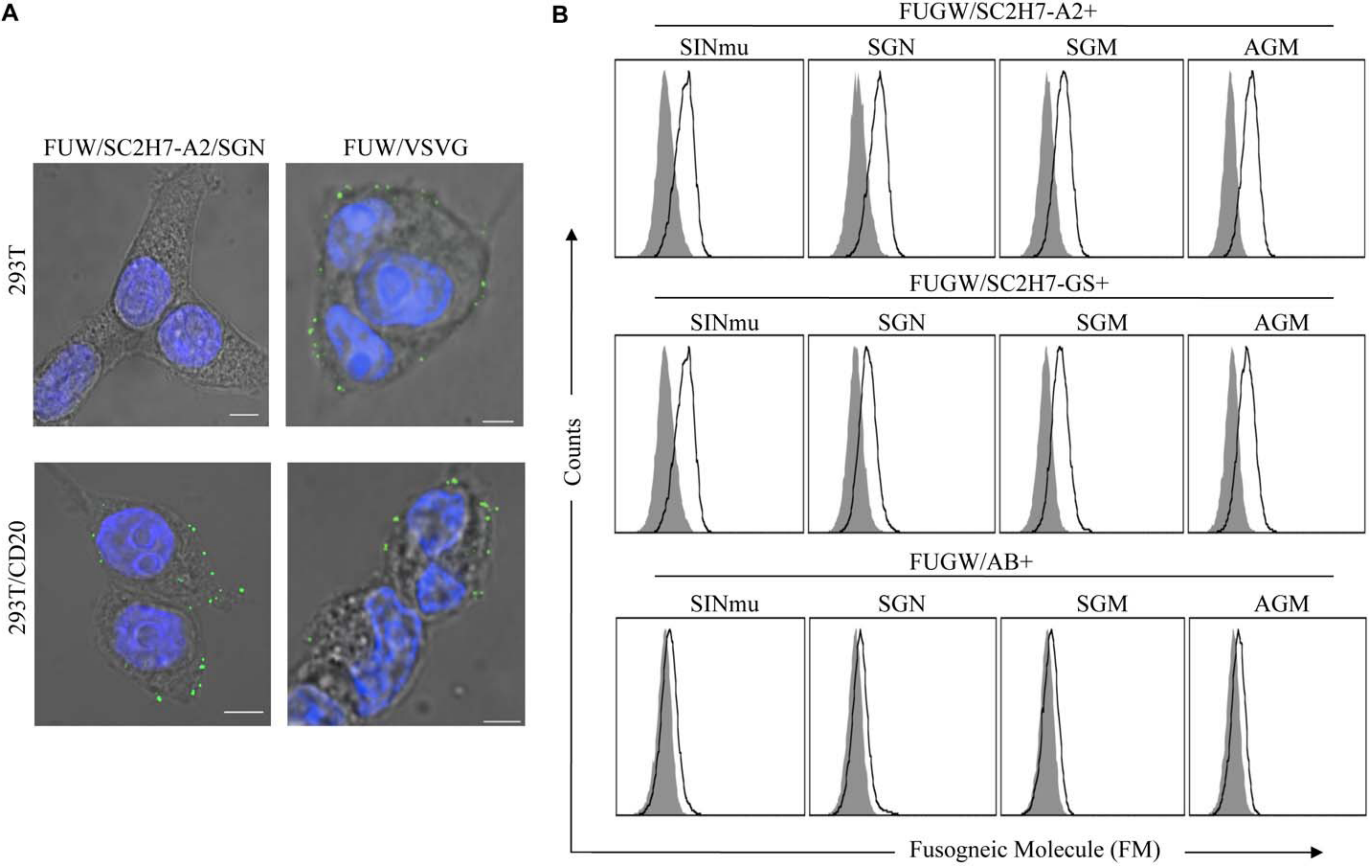
**Incorporation of both FM and antibody onto the vector surface**. (A) 293T (top) and 293T/CD20 (bottom) cells were incubated with either FUGW/SC2H7-A2/SGN (left) or FUGW/VSVG (right) at 4°C for 1 hour, fixed and immunostained with anti-p24 antibody (green) and DAPI nuclear staining (blue). Images were acquired with a laser scanning confocal microscope. Scale bar represents 2 μm. (B) Co-expression of antibody and FM on the same viral surface. 293T (shaded area) or 293T/CD20 (solid line) cells were incubated with FUGW/SC2H7-A2/FM, FUGW/SC2H7-GS/FM or FUGW/AB/FM at 4°C for 1 hour, followed by staining of FM by anti-HA antibody. The binding of the virus to the cells was detected by FACS analysis.

A virus-cell binding assay was performed to evaluate SCAb-mediated binding to CD20-expressing cells. As a target, we used a 293T cell line stably expressing the CD20 antigen (designated as 293T/CD20). The parental cell line 293T served as a negative control. The lentiviral vector, FUW/SC2H7-A2/SGN or FUW/VSVG, was incubated with either the target cell line, 293T/CD20, or the control cell line, 293T, for one hour at 4°C, after which, the cell-virus complex was fixed with 4% formaldehyde and stained by an anti-p24 antibody to detect the viral core and 4',6-diamidino-2-phenylindole (DAPI) for nucleus. As shown in Fig. 3A, confocal images revealed that the lentiviral vector (FUW/SC2H7-A2/SGN) was able to bind to 293T/CD20 cell line, but not to the control 293T cell line. In contrast, the lentiviral vector FUW/VSVG was able to bind to both 293T and 293T/CD20 cell lines. In addition, a quantitative virus-cell binding assay was conducted to evaluate SCAb-mediated binding to CD20-expressing cells. Lentiviral vectors (FUGW/SC2H7-A2/FM, FUGW/SC2H7-GS/FM, and FUGW/AB/FM) were incubated with either 293T or 293T/CD20 cells for one hour at 4°C to prevent internalization of the viral particle. Cells were then stained for the presence of viral particles on the cell surface using an anti-FM antibody and quantified using flow cytometry. As shown in Fig. 3B, flow cytometry analysis showed that the vector of either FUGW/SC2H7-A2/FM or FUGW/SC2H7-GS/FM was able to bind to 293T/CD20 cells. FACS analysis also showed that the virus bound to the 293T/CD20 cell surface displayed the FMs (Fig. 3B), suggesting that both SCAb and FM were incorporated on the same virion. Additionally, the control 293T cells showed no detectable FM, confirming that the observed viral particle binding to the cells is indeed due to the SCAb-antigen interaction. Similarly, a non-targeting lentiviral vector FUGW/AB/FM was unable to bind to either 293T or 293T/CD20 cells.

### Targeted transduction of lentiviral vectors

We conducted transduction experiments to evaluate the efficiency of lentiviral vectors bearing both SCAb and FM to transduce the CD20-expressing cell line. The lentiviral vector bearing VSVG was used as a positive control, whereas the lentiviral vector co-displaying AB and FM was included as a negative control. Cell lines were transduced by indicated lentiviral vectors and analyzed by FACS five days post-transduction. The GFP expression level was detected to quantify the specificity and efficiency.

**Figure 4:**
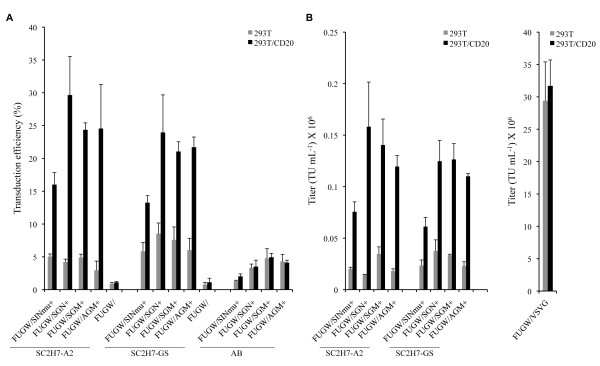
**Targeted transduction of lentiviral vectors to 293T/CD20 cells**. (A) 293T/CD20 (black bar) or 293T (grey bar) cells were transduced with 1.5 mL of fresh unconcentrated viral vectors (FUGW/SC2H7-A2/FM, FUGW/SC2H7-GS/FM, FUGW/AB/FM, FUGW/SC2H7-A2, or FUGW/SC2H-GS). FACS analysis was conducted to analyze the percentage of GFP-expressing cells 5 days post-transduction. (B) Transduction titers of fresh viral vectors (FUGW/SC2H7-A2/FM, FUGW/SC2H7-GS/FM, or FUGW/VSVG) on 293T (grey bar) and 293T/CD20 (black bar) cells.

Recombinant lentiviral vectors bearing both SCAb and FM were able to specifically transduce the 293T/CD20 cell line with various efficiencies (15% ~ 30%) varying upon the choice of the FMs (Fig. 4A). In contrast, less than 5% of transduction efficiency was observed for the 293T cell line. In addition, the titer of FUGW/SC2H7-A2/SGN was estimated to be ~0.15 × 10^6 ^transduction units (TU)/mL on the 293T/CD20 cells (Fig. 4B); the titer was determined in the dilution ranges that showed a linear response of GFP expression with viral serial dilution. In another control experiment, when the lentiviral vector bearing an isotype antibody paired with a FM (FUGW/AB/FM) were used, less than 5% of cells were transduced to express GFP. This finding further highlighted the significance of antibody-directed transduction. No transduction was observed with the lentiviral vector containing only SCAb, indicating the necessity of FM to complete transduction. Thus, lentiviral vectors must display both SCAb and FM for efficient transduction to target cells.

Among the various lentiviral vectors bearing the same SCAb but different FMs, different transduction efficiencies were observed. The lentiviral vector displaying SC2H7-A2 and SINmu exhibited 15% transduction efficiency. However, lentiviral vectors displaying other FMs (SGN, SGM, and AGM) resulted in specific transductions of 25% to 30%. A similar trend was observed in another independent study where the SCAb with VSVG transmembrane domain was used as the targeting antibody (SC2H7-GS) (Fig. 4A). In this case, the lentiviral vector bearing SINmu and SC2H7-GS was able to specifically transduce about 14% of the 293T/CD20 cells, whereas the specific transduction efficiency was increased to 25% when other FMs (SGN, SGM and AGM) were used in combination with the SC2H7-GS.

### Assays for studying the entry mechanism

**Figure 5:**
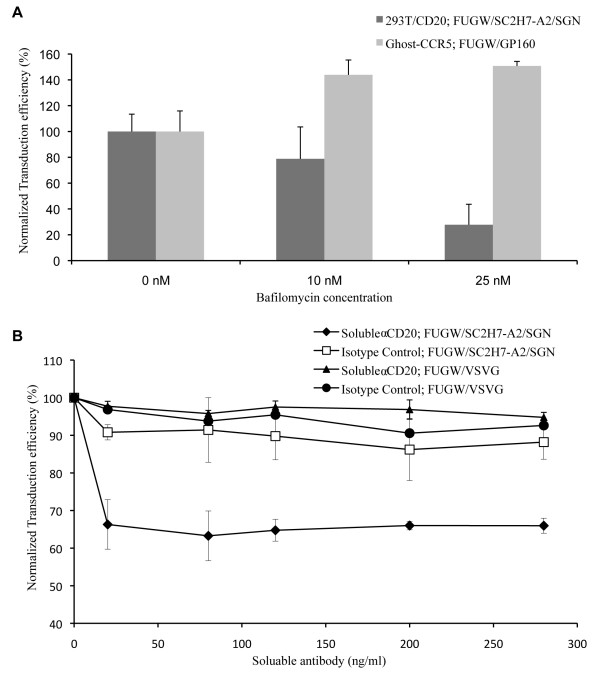
**Study of the entry mechanism of engineered lentiviral vector for transducing target cells**. (A) 293T/CD20 or Ghost-CCR5 cells were pre-incubated with various amount of bafilomycin for 30 minutes and spin-transduced with FUGW/SC2H7-A2/SGN or FUGW/GP160. Cells were incubated for an additional 3 hours before replenishing with fresh media. Transduction efficiency was measured by FACS analysis of GFP-positive cells 3 days post-transduction. All data was normalized to transduction without bafilomycin treatment. (B) Effects of supplement of soluble αCD20 on targeted transduction. 293T/CD20 cells were incubated with FUGW/SC2H7-A2/SGN or FUGW/VSVG and various amounts of soluble αCD20 or isotype control for 12 hours, after which the medium was replaced with fresh medium. Transduction efficiency was measured by FACS analysis of GFP-positive cells 3 days post-transduction. All data was normalized to transduction without soluble antibody treatment.

We hypothesized that our engineered lentiviral vector entered cells via receptor-mediated endocytosis followed by the endosomal fusion leading to the release of the vector core. To validate our hypothesis, we designed two independent experiments to study these two critical steps. Since the combination of SGN and SC2H7-A2 showed the greatest targeting efficiency in the transduction experiment, we chose this combination for the study of the entry mechanism. 293T/CD20 cells were exposed to either FUGW/SC2H7-A2/SGN or FUGW/VSVG in the presence of various amount of either soluble αCD20 or isotype control antibody (Fig. 5B). As expected, the level of transduction efficiency (FUGW/SC2H7-A2/SGN) dropped as the concentration of the soluble αCD20 increased, whereas no noticeable reduction in transduction efficiency was observed when the isotype control was used. In contrast, transduction efficiency of FUGW/VSVG was not affected by soluble αCD20.

The second critical step of transduction pathway involved the pH-dependent fusion event leading to the release of the viral core. To verify the pH requirement, we incubated either FUGW/SC2H7-A2/SGN or FUGW/GP160 with 293T/CD20 or Ghost-CCR5 cells in the increased presence of bafilomycin, which can raise the pH of the endosomal compartment. We observed a dramatic decrease in transduction efficiency (FUGW/SC2H7-A2/SGN) in response to increasing amount of bafilomycin (Fig. 5A). In a control experiment where a pH-independent virus (FUGW/GP160) was used, an increase in transduction efficiency was observed, which was consistent with previously published data [29,30]. Thus, the pH in the endosomal compartment is critical for viral membrane fusion.

### pH dependency study on the FMs

**Figure 6:**
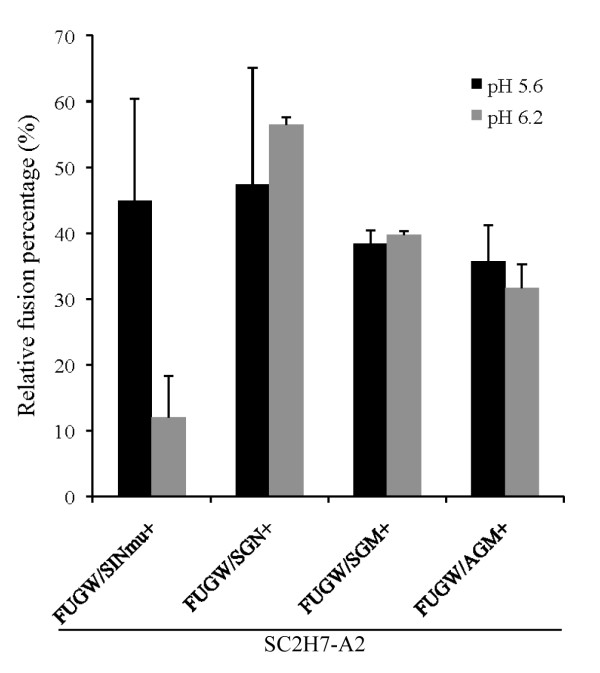
**pH-dependent study of the fusion activity of various FMs**. R18-labeled lentiviral vectors (FUGW/SC2H7-A2/FM) were mixed with liposomes (200 μM) for 1 minute. Virus-liposome fusion was triggered by adding the appropriate volume of acetic acid and measured by dequenching of fluorescent R18 using a spectrofluorometer.

As shown from the targeted transduction experiment, lentiviral vectors enveloped with various FMs resulted in different targeting efficiency (Fig. 4). We thus designed a liposome-virus fusion experiment to characterize the fusion property of these FMs. As shown in Fig. 6, roughly 40% to 50% of the lentiviral vector (FUGW/SC2H7-A2/FM) fused at pH of 5.6. When the same experiment was performed at pH environment of 6.2, only 12% of the lentiviral vector bearing SINmu fused, whereas a 40% to 50% fusion activity was obtained for vectors bearing other FMs (SGN, SGM, and AGM). Correlating the liposome-virus experiment with the targeted transduction experiment (Fig. 4), we observe a clear trend showing that the higher fusion activity of the FMs results in a higher transduction efficiency.

### Binding avidity of lentiviral vectors to target cells

**Figure 7:**
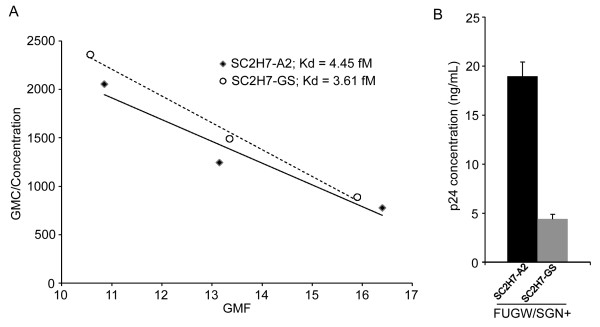
**Scatchard analysis of the lentiviral vector binding to 293T/CD20 cells**. (A) 293T/CD20 cells were incubated with various concentrations of lentiviral vectors (FUGW/SC2H7-A2/SGN or FUGW/SC2H7-GS/SGN) and stained with anti-HA antibody. The apparent K_d _value (1/slope) was derived from the scatchard plot of geometric mean fluorescence (GMF)/concentration versus GMF. (B) p24 concentration of lentiviral vectors (FUGW/SC2H7-A2/SGN and FUGW/SC2H7-GS/SGN).

In order to understand the different transduction efficiency of lentiviral vectors bearing these two different versions of SCAb (SC2H7-A2 and SC2H7-GS), we conducted a binding avidity assay. Increasing amount of the lentiviral vectors (FUGW/SC2H7-A2/SGN and FUGW/SC2H7-GS/SGN) were incubated with 293T/CD20 cells followed by the surface staining of the FM. The geometry mean fluorescence (GMF) intensity was measured and scatchard analysis was performed to determine the avidity of the lentiviral vector to bind to 293T/CD20 cells (Fig. 7A). In agreement with our transduction experiment (Fig. 4), the SC2H7-A2-enveloped lentiviral vector showed slightly better binding avidity to the target cells as compared to that of the SC2H7-GS-enveloped lentiviral vector. We also noted that when SC2H7-A2 was used to envelope the lentiviral vector, the vector production was increased as compared to that of SC2H7-GS (Fig. 7B). These findings explain the result of transduction experiment where the lentiviral vector pseudotyped with the SC2H7-A2 antibody showed higher transduction efficiency as compared to that of SC2H7-GS-bearing vector (Fig. 4).

## Discussion

The purpose of this study is to incorporate both membrane-bound SCAb and FM on the lentiviral surface to achieve targeted transduction to specific cell types. Previously, we reported a strategy of separating the binding and fusion functions of viral glycoprotein for cell specific targeting [21]. By pairing the αCD20 with a more fusion active FM, the resulted lentiviral vectors showed enhanced transduction [23]. In this study, we extended the targeting strategy to utilize a membrane-bound SCAb with the engineered FMs. Insertion of SCAb into the viral glycoprotein has shown to be able to redirect vector particles to specific cellular target [8,9,13]. However, these modifications usually resulted in reduced transduction efficiency. Our strategy of separating binding and fusion functions allows us to engineer a targeting lentiviral vector system by optimizing these two parameters in parallel without compromising their functions.

The lentiviral vectors bearing both SCAb and FM can specifically transduce CD20-expressing cells. The specific transduction occurs through a two-step process. First the virus must recognize and bind to CD20-expressing cells. Using flow cytometry and confocal microscopy, we verified that the SCAb was able to mediate the binding of the vector to the CD20 antigen on the cellular surface. Furthermore, the soluble αCD20 inhibition assay revealed that the targeting kinetics of the SCAb vector was inhibited in a dose-dependent fashion, confirming the binding requirement for the observed targeting. The second step for targeted transduction is the FM-mediated endosomal fusion to deliver the viral payload into the cell. A high titer and efficient transduction demonstrated that the FM was functional when combined with SCAb on the viral surface. Thus, the targeting lentiviral vector succeeds in these two steps to achieve efficient transduction.

As suggested from our previous studies, two different approaches can be applied to further optimize this two-molecule targeting strategy. By engineering the fusion loop of the SINmu, transduction can be enhanced [23]. Lentiviral vectors incorporating SCAb and SINmu consistently yielded lower transduction efficiency as compared to viral vectors with other FMs (SGN, SGM, or AGM). The difference in transduction efficiency may have resulted from the endosomal fusion kinetics of the different FMs. Recent studies of alphavirus glycoproteins have indicated that mutation in the E1 fusion domain might favor an increase in endosomal fusion ability [31-33]. We suspected that our mutation in the E1 domain might have a similar role in lowering the activation energy for the fusion event. Our liposome-virus fusion assay revealed that SINmu was not fusion-active at pH = 6.2, while other FMs were active at this pH. This direct correlation between the pH of fusion and the transduction efficiency suggests that the FMs that are more active at a higher pH can have better capacity to mediate lentiviral transduction. Consequently, targeted transduction may be further improved by constructing a library of FMs and screening for a FM with higher pH fusion activity.

Another approach to optimize this two-molecule targeting strategy is to engineer the targeting antibody to be more efficiently incorporated onto the lentiviral vector surface. Having the targeting molecule more efficiently incorporated onto the vector surface could enhance the binding of the vector to the cognate receptor on the target cell surface, thereby increasing transduction efficiency. To enhance the display of SCAb onto the viral surface, we constructed two SCAbs, each fused to a different transmembrane domain: the HLA-A2 transmembrane domain or the VSVG transmembrane domain. The targeted transduction efficiency was consistently higher with the SC2H7-A2-bearing vector. The binding avidity from the scatchard analysis revealed that the FUGW/SC2H7-A2/SGN vector exhibited a slightly higher binding avidity as compared to FUGW/SC2H7-GS/SGN. The higher avidity of the SC2H7-A2-bearing vector may be due to more efficient incorporation of SC2H7-A2 onto the lentiviral vector surface. It has been proposed that lipid rafts can serve as assembly sites for the pseudotyped lentiviral vectors [34]. Recent studies have demonstrated a correlation between transmembrane domain and raft association with efficient viral incorporation [35]. Although these data indicate a role of the transmembrane interaction to facilitate more efficient incorporation onto the virus, further understanding is needed to identify the precise mechanism of the transmembrane to facilitate incorporation of both the SCAb and FMs.

## Materials and methods

### Construct preparation

To generate the SCAb against the CD20 antigen, we first PCR-amplified the light chain variable region from an αCD20 hybridoma cell line (ATCC, Manassas, VA, HB-9803) with primers CD20Lvfw (5'-CTG ACC CAG ACC TGG GCG CAA ATT GTT CTC TCC CAG TCT CCA GCA ATC CTG TC-3') and CD20LvGSbw (5'-CAC CTC CTG AAC CAC CGC CGC TAC CGC CTC CGC CTT TCA GCT CCA GCT TGG TCC CAG CAC C-3'). The HLA-A2 leading peptide sequence was then added to the 5'-end of the light chain variable region with primers HLA-A2 (5'-GAA CAA TTT GCG CCC AGG TCT GGG TCA GGG CCA GAG CCC CCG AGA GTA GCA GGA CGA GGG TTC-3') and HLA-A2fw (5'-CTT AAG CTT ATG GCC GTC ATG GCG CCC CGA ACC CTC GTC CTG CTA CTC TCG GGG G-3'). We also PCR-amplified the heavy chain variable region with primers CD20hvGSfw (5'-GGT AGC GGC GGT GGT TCA GGA GGT GGC GGC AGT GGT GGA GGA TCT CAG GCT TAT CTA CAG CAG TCT GGG GCT GAG CTG-3') and CD20hvbw (5'-GTT TTG TCA CAA GAT TTG GGC TCA ACT GAA GAG ACG GTG ACC GTG GTC CCT GTG-3'). The PCR product was assembled with the light chain variable region using the primers HLA-A2fw and CD20hvbw. To fuse the hinge-CH2-CH3 domain to the HLA-A2 transmembrane domain, we PCR-amplified the hinge-CH2-CH3 domain and the HLA-A2 transmembrane domain using the primer pairs (CH2-CH3-Hingefw, 5'-GTC TCT TCA GTT GAG CCC AAA TCT TGT GAC AAA ACT CAC ACA TGC CCA CCG TGC CCA GCA CCT GAA CTC CTG GGG GGA CCG TC-3'; CH2-CH3bw, 5'-CTG GGA AGA CGG GGC CCC CTG TCC GAT CAT GTT CCT G-3') and (HLA-A2Tfw, 5'-GAC AGG GGG CCC CGT CTT CCC AGC CCA CCA TCC CC-3'; HLA-A2Tbw, 5'-CGA GCG GCC GCT CAC ACT TTA CAA GCT GTG AGA GAC ACA TCA GAG CCC-3'). The resulting two PCR fragments were assembled using primers CH2-CH3-Hingefw and HLA-A2Tbw. We then assembled the variable fragments with the CH2-CH3/transmembrane domain using the primers HLA-A2fw and HLA-A2Tbw. The assembled DNA was finally cloned into pcDNA3 (Invitrogen) via Hind3 and Not1 restriction sites. To construct a single chain antibody with the VSVG transmembrane domain, a forward primer (SC2H7fw, 5'-CCC CCA TCC CGG GAT GAG CTG ACC-3') and a backward primer (SC2H7bw, 5'-AGT ATC ACC GGC CCC CTG TCC GAT CAT GTT CCT GTA GTC-3') were used to amplify a portion of the CH2-CH3 domain of SC2H7-A2. In parallel, a forward primer (GSfw, 5'-ATG ATC GGA CAG GGG GCC GGT GAT ACT GGG CTA TCC AAA AAT CCA ATC GAG CTT-3') and a backward primer (GSbw, 5'-GAT CGA GCG GCC GCT TAC TTT CCA AGT CGG TTC ATC TCT ATG TCT GTA TAA ATC TGT CTT TTC-3') were used to amplify the transmembrane domain of VSVG. The DNA products from these two reactions were PCR-assembled using SC2H7fw and GSbw as the primer pair and the resulting product was cloned into pSC2H7-A2 to yield SC2H7-GS. The integrity of these constructs was confirmed by DNA sequencing.

### Viral vector production

293T cells were seeded in a 6-cm culture dish in DMEM medium supplemented with fetal bovine serum (Sigma, St. Louis, MO, 10%), L-glutamine (10 mL/L), penicillin, and streptomycin (100 units/mL) the night prior to transfection. 293T cells were transfected at a confluence of 80~90% with 5 μg of lentiviral backbone vector (FUGW), 2.5 μg each of pMDLg/pRRE, pRSV-Rev, pFM, and a plasmid encoding an antibody (pSC2H7-A2, pSC2H7-GS or pAB) via the standard calcium phosphate precipitation technique [36]. Cells were replenished with pre-warmed media 4 hours post-transfection. Vectors were harvested two days post-transfection and filtered through a 0.45-μm pore size filter (Nalgene, Rochester, NY). Lentiviral vectors were then further concentrated by ultracentrifugation (Optimal L-90K Ultracentrifuge, Beckman Coulter, Fullerton, CA) at 4°C, 25,000 rpm for 90 minutes and resuspended in appropriate volume of cold PBS.

### Virus-cell binding assay

293T/CD20 or 293T cells were incubated with 2 mL of lentiviral vectors (FUGW/SC2H7-A2/FM, FUGW/SC2H7-GS/FM or FUGW/AB/FM) at 4°C for 1 hour. After extensive washing with cold PBS, cell-virus complexes were stained with anti-HA tag antibody (Miltenyi Biotec, Inc.) and analyzed by flow cytometry (FACSort, BD Bioscience).

### Confocal imaging

Fluorescent images were acquired on a Zeiss LSM 510 META laser scanning confocal microscope equipped with Argon, red HeNe, and green HeNe lasers as well as a Coherent Chameleon Ti-Sapphire laser for multiphoton imaging. Images were acquired using a Plan-apochromat 63x/1.4 oil immersion objective. To image virus-cell binding, cells were seeded into a 35-mm glass-bottom culture dish and grown at 37°C overnight. The seeded cells were rinsed with cold PBS and incubated with concentrated viral particles for 1 hour at 4°C to allow for binding. The cells were washed with cold PBS to remove unbound particles, fixed with 4% formaldehyde on ice for 10 minutes, and then immunostained with monoclonal antibody specific for HIV capsid protein p24 and 4',6-diamidino-2-phenylindole (DAPI) antibody for nuclear staining. Monoclonal antibody against HIV-1 p24 (AG3.0) was obtained from the NIH AIDS Research and Reference Reagent Program (Division of AIDS, NIAID, NIH). Images were analyzed using the Zeiss LSM 510 software version 3.2 SP2.

### Antibody Competition Assay

293T/CD20 cells were incubated with the lentiviral vector (FGUW/SC2H7-A2/SGN or FUGW/VSVG) and various amount of either the soluble αCD20 (BD Bioscience) or the isotype control antibody overnight. Cells were then replenished with fresh media and incubated for additional 72 hours before flow cytometry analysis.

### Neutralization Assay

293T/CD20 or Ghost-CCR5 (NIH AIDS Research and Reference Reagent Program) cells were pre-incubated with various amount of bafilomycin for 30 minutes, after which, the lentiviral vector (FUGW/SC2H7-A2/SGN or FUGW/GP160) was added. The vector and cell mixture was spun at 25°C, 2,500 rpm for 90 minutes using a RT legend centrifuge (Sorval). Cells were then incubated at 37°C and 5% CO_2 _and replenished with fresh media 3 hours later. Flow cytometry was then used to analyze the treated cells 3 days post-transduction.

### Targeted transduction of 293T/CD20 cells

293T or 293T/CD20 cells were seeded on a 24-well cell culture plate and spin-transduced with 1.5 mL of indicated lentiviral vectors (FUGW/SC2H7-A2/FM, FUGW/SC2H7-GS/FM, FUGW/AB/FM, FUGW/SC2H7-A2, or FUGW/SC2H7-GS) at 25°C, 2,500 rpm for 90 minutes using a RT legend centrifuge. After replacing with fresh media, the treated cells were cultured for additional 5 days at 37°C and 5% CO_2_. Flow cytometry was then used to analyze transduction efficiency. The titer was determined by measuring GFP-positive cells in the dilution range that resulted in a linear relationship between the percentage of GFP-expressing cells and the amount of vectors added.

### Scatchard analysis

293T/CD20 cells were incubated with various amount of lentiviral vectors (FUGW/SC2H7-A2/SGN or FUGW/SC2H7-GS/SGN). Flow cytometry analysis was carried out to measure the geometric mean fluorescence (GMF) of the bound viruses stained by anti-HA antibody. The concentration of the lentiviral vectors was measured by a p24 antigen capture enzyme immunosorbent assay (ELISA) kit (ImmunoDiagnostics, Woburn, MA). Apparent K_d _value was derived from the negative reciprocal of the slope of the linear fit to scatchard plots, which is the geometric mean fluorescence/concentration of lentiviral vector (GMF/concentration) against geometric mean fluorescence (GMF).

### Virus-liposome fusion assay

1,2-dioleoyl-sn-glycero-3-phosphocholine (DOPC) and 1,2-dioleoyl-sn-glycero-3-phosphoethanolamine (DOPE) were purchased from Avanti Polar Lipids (Alabaster, AL, USA). Cholesterol (Chol) and sphingomyelin (SPM) from egg yolk were obtained from Sigma (St Louis, MO, USA). Liposomes were prepared by the extrusion procedure [37]. Briefly, lipid mixtures (PC/PE/SPM/Chol molar ratio of 1:1:1:2) were dried from a chloroform solution under a stream of argon gas and further dried under vacuum for at least 3 hours. The lipid mixtures were hydrated in HNE buffer (5 mM HEPES, 150 mM NaCl, and 0.1 mM EDTA, pH 7.4). Subsequently, the lipid mixtures were extruded 20 times through 0.2 μm pore size polycarbonate filters (Avanti polar lipids). To monitor virus-liposome fusion, the concentrated viruses were incubated with 70 μM of octadecyl rhodamine B chloride (R18) (Molecular Probes, Carlsbad, CA, USA) in serum-free medium for 1 hour at room temperature. R18-labeled viruses were then mixed with liposomes (200 μM phospholipids) in a final volume of 0.4 mL. Fusion was triggered by adding the appropriate volume of 0.2 M acetic acid, pretitrated to achieve the desired pH. The dequenching signal of R18 fluorescence was measured 60 seconds after acidification with QuantaMaster QM-4SE spectrofluorometer (Photon Technology International, Lawrenceville, NJ, USA). The initial fluorescence of virus-liposome mixtures was set at 0% fusion, and the 100% fusion value was obtained by detergent lysis for each experiment using 0.1% of Triton X-100 [38].

## Competing interests

The authors declare that they have no competing interests.

## Authors' contributions

YL and PW designed research; YL, KJ, JZ and CW performed research; YL and PW analyzed data and wrote the paper. All authors read and approved the final manuscript.
